# Uncovering the Complexity of Perinatal Polysubstance Use Disclosure Patterns on X: Mixed Methods Study

**DOI:** 10.2196/53171

**Published:** 2024-09-20

**Authors:** Dezhi Wu, Hannah Shead, Yang Ren, Phyllis Raynor, Youyou Tao, Harvey Villanueva, Peiyin Hung, Xiaoming Li, Robert G Brookshire, Kacey Eichelberger, Constance Guille, Alain H Litwin, Bankole Olatosi

**Affiliations:** 1 Department of Integrated Information Technology University of South Carolina Columbia, SC United States; 2 Department of Mathematics Augusta University Augusta, GA United States; 3 Department of Computer Science and Engineering University of South Carolina Columbia, SC United States; 4 College of Nursing University of South Carolina Columbia, SC United States; 5 Department of Information Systems and Business Analytics Loyola Marymount University Los Angeles, CA United States; 6 Arnold School of Public Health University of South Carolina Columbia, SC United States; 7 School of Medicine Greenville University of South Carolina Greenville, SC United States; 8 Prisma Health Greenville, SC United States; 9 College of Medicine Medical University of South Carolina Charleston, SC United States

**Keywords:** polysubstance use, prenatal care, perinatal care, pregnant care, social media, Twitter, sentiment analysis

## Abstract

**Background:**

According to the *Morbidity and Mortality Weekly Report*, polysubstance use among pregnant women is prevalent, with 38.2% of those who consume alcohol also engaging in the use of one or more additional substances. However, the underlying mechanisms, contexts, and experiences of polysubstance use are unclear. Organic information is abundant on social media such as X (formerly Twitter). Traditional quantitative and qualitative methods, as well as natural language processing techniques, can be jointly used to derive insights into public opinions, sentiments, and clinical and public health policy implications.

**Objective:**

Based on perinatal polysubstance use (PPU) data that we extracted on X from May 1, 2019, to October 31, 2021, we proposed two primary research questions: (1) What is the overall trend and sentiment of PPU discussions on X? (2) Are there any distinct patterns in the discussion trends of PPU-related tweets? If so, what are the implications for perinatal care and associated public health policies?

**Methods:**

We used X’s application programming interface to extract >6 million raw tweets worldwide containing ≥2 prenatal health- and substance-related keywords provided by our clinical team. After removing all non–English-language tweets, non-US tweets, and US tweets without disclosed geolocations, we obtained 4848 PPU-related US tweets. We then evaluated them using a mixed methods approach. The quantitative analysis applied frequency, trend analysis, and several natural language processing techniques such as sentiment analysis to derive statistics to preview the corpus. To further understand semantics and clinical insights among these tweets, we conducted an in-depth thematic content analysis with a random sample of 500 PPU-related tweets with a satisfying κ score of 0.7748 for intercoder reliability.

**Results:**

Our quantitative analysis indicates the overall trends, bigram and trigram patterns, and negative sentiments were more dominant in PPU tweets (2490/4848, 51.36%) than in the non-PPU sample (1323/4848, 27.29%). Paired polysubstance use (4134/4848, 85.27%) was the most common, with the combination *alcohol and drugs* identified as the most mentioned. From the qualitative analysis, we identified 3 main themes: *nonsubstance*, *single substance*, and *polysubstance,* and 4 subthemes to contextualize the rationale of underlying PPU behaviors: *lifestyle*, *perceptions of others’ drug use*, *legal implications*, and *public health*.

**Conclusions:**

This study identified underexplored, emerging, and important topics related to perinatal PPU, with significant stigmas and legal ramifications discussed on X. Overall, public sentiments on PPU were mixed, encompassing negative (2490/4848, 51.36%), positive (1884/4848, 38.86%), and neutral (474/4848, 9.78%) sentiments. The leading substances in PPU were *alcohol and drugs*, and the normalization of PPU discussed on X is becoming more prevalent. Thus, this study provides valuable insights to further understand the complexity of PPU and its implications for public health practitioners and policy makers to provide proper access and support to individuals with PPU.

## Introduction

### Background

There is a growing concern regarding the rising number of pregnant women who use harmful substances [1]. According to the National Survey on Drug Use and Health, 8.5% of pregnant women in the United States reported illicit drug use, 7.1% reported marijuana use, and 11.5% consumed alcohol in the previous month [2]. These figures represent a significant increase since 2015, with a 4.7% rise in illicit drug use, a 3.4% increase in marijuana use, and a 9.3% surge in alcohol use [3]. It is even more alarming that the incidence of polysubstance use disorders (PUDs) increases during the perinatal period, with pregnant women consuming more than one harmful substance. The *Morbidity and Mortality Weekly Report* highlights that 38.2% of pregnant individuals who consumed alcohol also reported using other substances [1]. In addition, nearly half of pregnant individuals who reported opioid use also disclosed alcohol consumption [4]. Thus, perinatal polysubstance use (PPU) poses severe risks to both mothers and babies, resulting in a significant public health issue and adverse effects for both mother and baby health at large.

Clinically, use of 1 substance during pregnancy can cause adverse health outcomes; for example, pregnant individuals who use tobacco or alcohol can already cause premature birth or low birth weight, respectively. Worse, the combined use substantially increases the risk of negative fetal outcomes [5]. Similarly, the co-use of opioids and alcohol during the perinatal period can lead to neonatal withdrawal syndrome, which manifests in infants as tremors, fever, and seizures [6], leading to suboptimal pregnancy outcomes [7]. Recent trends show an alarming increase in harmful PPU associated with severe fetal health effects such as birth defects, delayed fetal development, and fetal demise [6,8]. These persistent maternal and fetal health challenges underscore the urgent need for public health policies, targeted interventions, and treatments designed to reduce and eliminate substance use during the pregnancy period and beyond [1,8].

Previous studies exploring substance use during pregnancy have primarily used interview data or survey data collected from national health samples [1,9-11]. However, a significant limitation of these traditional approaches is that pregnant women might withhold information due to feelings of shame or guilt, especially if they fear judgment from interviewers. For example, Paris et al [11] delved into interviews to understand why pregnant women conceal their substance use and what eventually prompts them to seek help. They found that shame, guilt, and societal stigma are primary factors leading women to hide their substance use from their providers when seeking prenatal care [11]. The stigma associated with substance use during pregnancy might prevent some participants from fully disclosing their reasons for continued drug use, which could exacerbate feelings of shame and both internal and external stigma [11]. These stigmas can reinforce secretive substance use patterns, delaying help-seeking behavior and potentially worsening health outcomes for both mother and child. In addition, the fear of facing critical charges may compel women to remain silent [12]. In this study context, compared to traditional data collected from interviews or surveys, social media could provide a more unfiltered platform for public discussions as individuals may choose to anonymously share their thoughts and concerns, potentially providing richer and more authentic data.

The widespread use of social media has transformed platforms such as X, formerly known as Twitter, into rich repositories of organic data that are not typically accessible through clinical trials, surveys, or interviews in the health care sector. As a result, these social media platforms have begun to function as public health surveillance tools, offering a new and vast source of big data for innovative research [13-15]. This shift has significantly enhanced our understanding of health issues and public opinions [13]. Social media data not only are massive but also offer the advantage of reducing biases that are often present in traditional interview-based studies [16]. Analyzing public opinions on social media has proven valuable to clinicians, public health officials, and policy makers, helping gauge the accuracy of health messages being disseminated and understand public perceptions of these messages, thereby informing more effective and relevant health communication strategies [14,15].

Extensive studies have explored the intersection of substance use disorder (SUD) and social media. For instance, research has identified a trend of normalization in tweets related to hookah use [17]. Tobacco-related content on social media propagates rapidly through social networks, especially when shared by popular users with large numbers of followers [18]. Furthermore, discussions related to health risks and warnings associated with nicotine products have been prominently featured on platforms such as X [19]. More specifically, social media has been proven to be an invaluable resource for gathering insights on substance use among pregnant individuals, providing a rich vein of user-generated content for further studies [20].

Despite the prevalence of social media for studying SUD, PPU patterns on social media are largely unexplored. This study sought to address this significant research gap by exploring and identifying PPU patterns among pregnant individuals to inform public health policies and clinical implications for perinatal care. Specifically, we planned to collect data on PPU from X, a social media platform that health care researchers constantly use for health-related data extraction and exploring real-time public health surveillance, different diseases, and web-based communities. In this study, we focused on identifying the most commonly discussed types of polysubstance use in perinatal care, and initially understanding what combinations of substances are more prevalent, why, and their public sentiments.

To achieve this, we began with a systematic investigation of PPU by analyzing tweets from X. We then aimed to uncover emerging topics and trends surrounding PPU in pregnant individuals and analyze sentiments. We collected a sample of 4848 US-based PPU tweets on X and used natural language processing (NLP) for frequency, trend, bigram, trigram, and sentiment analyses. Using another sample as a control group with non-PPU tweets during the same time frame, we compared the PPU and non-PPU groups and identified significant differences, allowing us to assess the accuracy of machine learning (ML) methods in sentiment analysis. To explore more in-depth themes and subthemes discussed in the PPU tweets, we applied qualitative content analysis using a random sample of 500 PPU tweets and identified multiple PPU themes and subthemes. As one of the first studies of its kind, we expect that our findings will offer valuable and timely insights for enhancing maternal health– and substance-related public health policies and PPU interventions more customized for the perinatal population.

### Objectives

In this study, we aimed to address the following two primary research questions (RQs):

1. What is the overall trend and sentiment of PPU discussions on X? (RQ 1)
2. Are there any distinct patterns in the discussion trends of PPU-related tweets? If so, what are the implications for perinatal care and associated public health policies? (RQ 2)

## Methods

### Data

Using the streaming application programming interface provided by the X Research Developer Platform, we initially extracted >6 million raw perinatal SUD–related tweets from around the world spanning May 1, 2019, to October 31, 2021. Subsequently, we refined this user-generated content by filtering out non–English-language tweets and tweets from non-US accounts and accounts without disclosed geolocations. In addition, we screened the data set to eliminate spam, advertisements, URLs, punctuation, stop words, and duplicate tweets. To be included in our analysis, each tweet had to contain at least 2 words from a predetermined data set of substance- and prenatal and child health–related keywords provided by our clinical team (detailed in Multimedia Appendix 1). After this rigorous filtering process, the final sample of relevant and clean tweets for this study comprised 4848 PPU-related tweets. To identify variances in our data, we also collected another set of 4848 non-PPU tweets on X during the same time frame as the control group for comparison analysis.

### Ethical Considerations

The University of South Carolina’s institutional review board reviewed our application (Pro00122484), social media data exaction, and analysis method for this study. This study was deemed non–human subjects research and thus exempted from review.

### Study Design

#### Overview

This study used a mixed methods approach to analyze PPU data from X. The Quantitative Analysis Methods section introduces the quantitative analysis methods applied in this study, whereas the Qualitative Analysis Methods section details the qualitative methods used. For the quantitative analysis, 4848 tweets were analyzed. From this data set, 500 PPU tweets were randomly selected for more in-depth qualitative analysis.

#### Quantitative Analysis Methods

The quantitative analysis leveraged NLP techniques to elucidate the attributes and characteristics of the corpus of tweets. We used 2 key NLP techniques: frequency analysis to understand tweet trends, bigrams, trigrams, and sentiment analysis for the PPU-related sample of 4848 tweets, which we also used to compare their sentiment differences against a control group using a set of random 4848 non-PPU tweets extracted from X during the same period [21,22].

Before applying NLP techniques, proper cleaning was performed on the tweet data set to ensure the accuracy of the NLP outcomes. This data cleaning was solely performed for the quantitative analysis to apply various ML algorithms to analyze our tweets. The process involved tokenizing cleaned tweets into arrays in which each word represented an element. We removed stop words such as *a*, *is*, and *the*, which offer limited insights into tweet sentiment. To avoid the potential loss of sentiment intensity when lemmatizing words, we used the raw text as input for our sentiment analysis. In addition, non–American Standard Code for Information Exchange characters were removed from the text.

Sentiment analysis, which classifies the expressed sentiment in text, was conducted using the Valence Aware Dictionary and Sentiment Reasoner (VADER), which is designed to account for nuances such as punctuation, capitalization, and even emojis, thus providing a more accurate reflection of sentiment in the text. VADER, a lexicon and rule-based tool tailored for social media, assesses the polarity (positive, negative, and neutral) of each tweet and assigns a sentiment score ranging from –1 to 1. For instance, in the tweet “I hate and discourage the use of drugs while pregnant,” it would highlight “hate” and “discourage” as negative, whereas in “I enjoy drugs and alcohol,” it would identify “enjoy” as positive. On the basis of established thresholds, tweets with scores of >0.05 are deemed positive, tweets with scores of <–0.05 are deemed negative, and those in between are deemed neutral [23]. Sentiment analysis has broad applications, including in marketing, politics, and health care, and is particularly effective in X-based health research [21,22]. Thus, in this study, we aimed to apply VADER sentiment analysis to identify the prevalent sentiments associated with the most frequently reoccurring substance combinations in PPU-related tweets.

Furthermore, we conducted frequency analyses, focusing on trend, bigram, and trigram analyses. Bigrams are sets of 2 words together, and trigrams are sets of 3 words together. This approach sheds light on prevalent discussion topics within the data set without necessitating a manual review of each tweet; as noted by Sarker et al [14], such analysis provides meaningful insights into the textual content.

#### Qualitative Analysis Methods

Qualitative analysis enabled a deeper exploration of the underlying semantics of individual tweets. By applying thematic content analysis, we gained deeper insights into PPU-related themes, subthemes, and user behaviors. This method, recently applied in the health care field, helps distill textual data into coherent themes. Thematic analysis emphasizes emergent themes and subjectivity, whereas content analysis focuses on explicit content and objectivity using either inductive or deductive approaches. Inductive content analysis derives concepts, categories, and themes directly from the data, whereas deductive analysis uses predefined frameworks [24,25].

For this analysis, we selected a random sample of 500 PPU tweets from our 4848 PPU-related tweets. In total, 2 independent researchers conducted the qualitative analysis using inductive coding techniques. The tweets were categorized into themes and subthemes using the NVivo software (version 12.0; Lumivero). The researchers started coding tweets together to ensure similar thought processes, followed by independent coding interspersed with periodic meetings to reconcile differences and jointly analyze more complex tweets. After coding all 500 tweets, we assessed interrater reliability using the κ score to determine the degree of agreement between the 2 coders and account for the probability of coding similarly by chance [26]. We computed 2 κ scores using NVivo’s built-in functions: one for the tweets in the main themes and the other for substance combinations. The resulting average κ score was 0.6940 for differentiating tweets about single-substance use, polysubstance use, and non–substance use, achieving reasonable agreement between the 2 coders. Further investigation was conducted to find the average κ score between the combinations of substances. For substance combinations, the average κ score was 0.7748, reflecting a satisfying agreement given that previous studies have identified a κ score of 0.60 as moderate [26] and 0.79 as significant [14].

**Figure 1:**
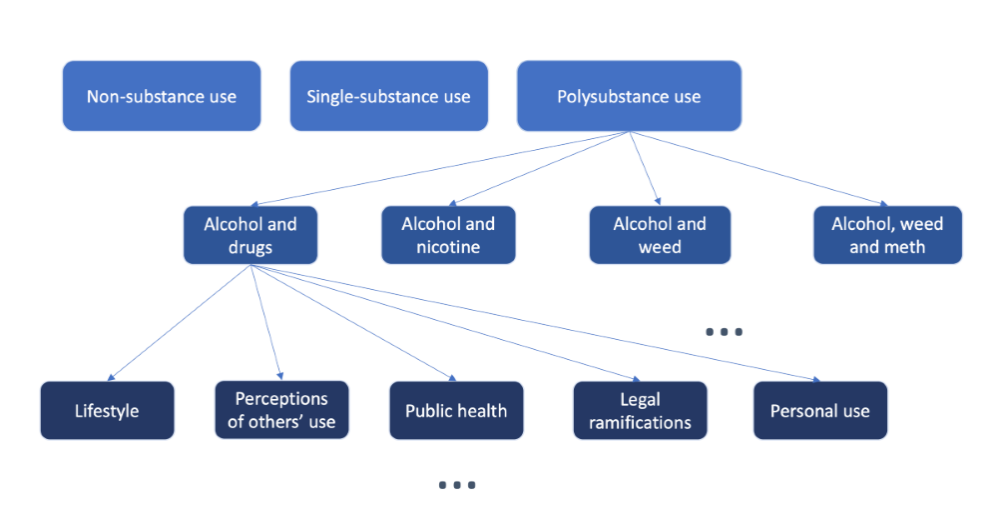
The general structure of the codebook. The 3 dots indicate more coding subbranches horizontally and vertically.

We established a codebook for this thematic content analysis. Figure 1 illustrates our coding structure with the first few layers of the multilayer codebook. Each tweet was first classified as non–substance use, single-substance use, or polysubstance use. Tweets regarding polysubstance use were further categorized by the specific combination of substances discussed. The final coding structure contains >3 levels or subbranches, so Figure 1 visually represents the general coding process for our qualitative analysis.

## Results

### Quantitative Analysis

#### Sentiment Analysis

To identify specific combinations of PPU, we analyzed commonly mentioned substances from the PPU tweets. We extracted combinations of substances that were both mentioned explicitly in a tweet; for instance, a tweet containing the words “alcohol” and “drug” was categorized into the “alcohol and drugs” combination. We then conducted sentiment analysis on the most frequently reoccurring substance combinations in our data. We further quantified sentiment differences using the chi-square test for the frequency counts of sentiment categories—positive, neutral, or negative—across PPU and non-PPU tweets. This statistical test was crucial for identifying significant variations in sentiment distributions between the 2 groups.

Table 1 displays the overall VADER sentiment analysis results for PPU tweets and random non-PPU tweets. To assess the sentiment distribution differences between PPU and non-PPU tweets, we applied the chi-square test and reported the results in Table 1. Notably, PPU tweets had a significantly lower percentage of positive sentiments (1884/4848, 38.86%) compared to the non-PPU tweet sample (2206/4848, 45.5%), with a *P* value of <.001, indicating that positive sentiments are less prevalent in PPU tweets. Conversely, negative sentiments were more common in PPU tweets (2490/4848, 51.36%) than in the non-PPU sample (1323/4848, 27.29%), with the statistical significance underscored by a *P* value of <.001. Neutral sentiments were significantly rarer in PPU tweets (474/4848, 9.78%) as opposed to in the control sample (1319/4848, 27.21%), also with a *P* value of <.001, suggesting a lower occurrence of neutral sentiments deemed useful for PPU analysis.

**Table 1:** Sentiment comparison analysis between perinatal polysubstance use (PPU) and non-PPU tweets (N=4848).

Sentiment	PPU tweets, n (%)	Random non-PPU tweets, n (%)	*P* value
Negative	2490 (51.36)	1323 (27.29)	<.001
Positive	1884 (38.86)	2206 (45.5)	<.001
Neutral	474 (9.78)	1319 (27.21)	<.001

Table 2 presents the sentiment analysis for the top 5 most reoccurring substance combinations. Regarding “alcohol and drugs,” “meth and drugs,” “weed and drugs,” and “cocaine and drugs,” the sentiment of over half of these substance pairs was negative, and the sentiment of <40% was positive, except for the sentiment of the “weed and meth” combination, which was mixed (positive: 70/160, 43.7%; negative: 76/160, 47.5%; neutral: 14/160, 8.8%).

**Table 2:** Sentiment analysis for the top 5 substance combinations.

Combination	Positive, n (%)	Neutral, n (%)	Negative, n (%)
Alcohol and drugs (n=861)	318 (36.9)	53 (6.2)	490 (56.9)
Meth and drugs (n=301)	122 (40.5)	14 (4.7)	165 (54.8)
Weed and drugs (n=202)	73 (36.1)	24 (11.9)	105 (52)
Weed and meth (n=160)	70 (43.7)	14 (8.8)	76 (47.5)
Cocaine and drugs (n=152)	51 (33.6)	11 (7.2)	90 (59.2)

#### Trend, Bigram, and Trigram Analyses

**Figure 2:**
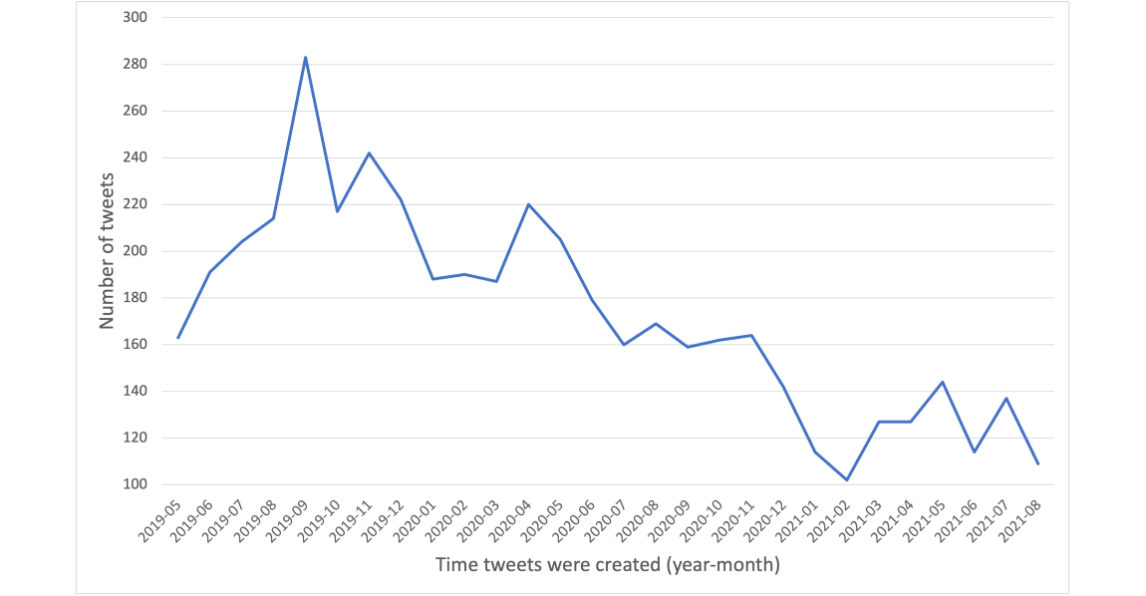
Overall trend of perinatal polysubstance use tweets from May 1, 2019, to October 31, 2021.

Next, we illustrate the overall tweet distribution of our main PPU tweets from May 2019 to October 2021 in Figure 2. We further present the frequency of the most popular bigrams and trigrams in PPU tweets (ie, 2- and 3-word sequences of words, respectively) in Table 3 and 4. Regarding the bigram frequencies listed in Table 3, the 2 apparent themes pertain to drug use and family dynamics. The most common bigrams were “drug alcohol,” “drug use,” and “smoke weed.” These bigrams provided a glimpse of the major substance combinations that we were able to further identify through qualitative analysis, discussed in the next section. In addition, there was a multitude of bigrams alluding to domestic situations (eg, “family member” and “child abuse”). Regarding the trigrams listed in Table 4, the most common ones were related to drug abuse. A large quantity of the results were trigram permutations of the words “drug,” “alcohol,” “abuse,” and “addiction.” Table 4 presents these polysubstances containing the dominant substances.

**Table 3:** Top 10 frequent bigrams in perinatal polysubstance use tweets.

Bigram	Frequency, n (%)
“Drug alcohol”	419 (23.92)
“Drug use”	356 (20.32)
“Smoke weed”	183 (10.45)
“Alcohol drug”	159 (9.08)
“Family member”	130 (7.42)
“Drug addict”	129 (7.36)
“Drug dealer”	102 (5.82)
“Drug user”	96 (5.48)
“Child abuse”	91 (5.19)
“Drug addiction”	87 (4.97)

**Table 4:** Top 10 frequent trigrams in perinatal polysubstance use tweets.

Trigram	Frequency, n (%)
“Drug alcohol abuse”	41 (17.52)
“Abuse drug alcohol”	24 (10.26)
“Alcoholic drug addict”	24 (10.26)
“Drug addict alcoholic”	23 (9.83)
“Child abuse drug”	22 (9.4)
“Mom pop vape”	22 (9.4)
“Drug alcohol addiction”	21 (8.97)
“Smell like weed”	20 (8.55)
“Alcoholism drug addiction”	19 (8.12)
“Fetal alcohol syndrome”	18 (7.69)

Overall, among the 4848 PPU tweets, we found a complicated polysubstance use among pregnant individuals. Of the 4848 tweets, 4134 (85.27%) tweets contained 2 substance keywords, 539 (11.12%) included 3 substance keywords, and 175 (3.61%) mentioned >3 substances. Most PPU patterns turned out to be paired with 2-substance use. The top 5 substance combinations were *alcohol and drugs*, *meth and drugs*, *weed and drugs*, *cocaine and drugs*, and *alcohol and meth*, listed in Table 2. Table 5 further details the percentages of tweets ranked as the top 11 substance combinations. Figure S1 in Multimedia Appendix 2 visually presents the overall substance combinations in the PPU tweets. Table S1 in Multimedia Appendix 2 further summarizes the top 60 substance combinations included in the “other” category, including other identified double and triple substance combinations and their percentage distributions in this study. The *alcohol and drugs* combination was the most frequently reoccurring polysubstance group. This further supports the common perceptions about alcohol’s popularity and its widespread acceptance, which has transformed general opinions to no longer consider it as a drug, as indicated in the following tweet quote, also indicating that legal substances tend to be discussed more openly and frequently on social media:

LMAO ALCOHOL AND TOBACCO IS LEGAL SO ARE YOU GONNA THINK THATS OKAY TO DO WHILE PREGNANT TOO?

**Table 5:** Percentage of the top 11 substance pairs identified in the perinatal polysubstance use tweets (N=4848).

Keyword 1	Keyword 2	Tweets, n (%)
Alcohol	Drugs	861 (17.76)
Meth	Drugs	301 (6.21)
Weed	Drugs	202 (4.17)
Weed	Meth	160 (3.3)
Cocaine	Drugs	152 (3.14)
Alcohol	Meth	146 (3.01)
Alcohol	Weed	130 (2.68)
Heroin	Meth	117 (2.41)
Heroin	Drugs	102 (2.1)
Opioid	Drugs	92 (1.9)
Weed	Cigarette	81 (1.67)

### Qualitative Analysis

#### Main Themes

We randomly selected a sample of 500 PPU tweets for a more in-depth thematic analysis. The tweets were initially coded into 3 categories depending on the substances mentioned (ie, *nonsubstance*, meaning that no substance was mentioned in the tweets; single substance, indicating that only 1 substance was discussed in the tweets; and *polysubstance*, representing multiple substances mentioned in tweets). PPU tweets were categorized based on the substance name and combination with another substance. We used 3 coding levels to provide an overall visual structure of our codebook illustrated in Figure 1 in the Qualitative Analysis Methods section, and we found more subthemes within each category to allow the researchers to capture the exact essence of each tweet.

Table 6 contains the definitions for each identified theme category with example tweets. Following our coding process (Figure 1), the 2 researchers independently coded and identified themes and subthemes for the sample of 500 PPU-related tweets. There may be some overlaps in the categories; for example, a tweet can start discussing single-substance use and then later discuss polysubstance use. We coded this type of tweets as polysubstance use because they discussed both drug and alcohol use.

**Table 6:** The 3 main themes discussed in the perinatal polysubstance use tweets.

Theme of substance use	Theme definition	Sample tweets
Non–substance use (n=36)	Content discussing the users’ opinions explicitly not related to substance use	“Sometimes I feel my fur child is a human! She drug her cover over to the couch, then got up and got under it https://link” “My children from 26 -35 yrs old were exposed to glyphosate from birth. Many rural children were. I wish they hadn’t been and we had not believed NZ and US weed scientists who assured it was safe enough to drink.”
Single-substance use (n=223)	Content discussing single-substance use	“I’m taking the Alcohol people out—are you pro-choice when it comes to abortion and drugs?” “Babies born to mothers who smoke tobacco are at greater risk for ADHD. Children of mothers with high levels of nicotine exposure were more than twice as likely to develop ADHD than children born to mothers with low levels of exposure.”
Polysubstance use (n=260)	Content discussing the use of multiple substances	“I’m not going to share my story (science speaks for itself), but #youknowme for sure. I abused drugs and alcohol for years on & off—really nasty ones that do terrible damage to a fetus. Poor nutrition, no medical care, & could not stop using even when I suspected I was pregnant.” “As a case worker I have had children look me in the eyes & ask me why their mommy & daddy don’t love them as much as they love drugs & alcohol. No child should have to search their entire life for someone to love them they way they deserved love from the beginning #ProChoice”

#### Substance Combinations

In addition to main-level categories or themes, we further coded the contents into subthemes to better illustrate the discussions on X. For the main PPU themes identified, we delved into specific drug combinations observed in the tweets. The most frequently reoccurring combinations were *alcohol and drugs*, *alcohol and cannabis*, and *alcohol and nicotine*. Table 7 presents a brief description of each, agreed upon by the 2 coders, along with example tweets. The κ score for the substance combinations was 0.7748.

**Table 7:** Definitions and examples of the most frequently reoccurring substance combinations in the perinatal polysubstance use tweets.

Combination subtheme	Definition	Sample tweets
Alcohol and drugs (n=120)	Discussions pertaining to the use of multiple substances or the mention of multiple substances in the same tweet	“i literally hate today because my father abandoned me for drugs, alcohol & hookers he was NEVER in my life he was always in and out of jail he stole money from my mom he never cared but NOW he’s coming back into my life & it sucks because i wish he were there when i needed him.” “The worst thing is watching someone you love throw their life away. Will never understand people who choose alcohol and drugs over their family and you can’t help them anymore. Some people don’t know how lucky they are.”
Alcohol and cannabis (n=26)	Discussions pertaining to the use of multiple substances or the mention of multiple substances in the same tweet	“I once read a forum where a guy was advocating weed to treat alcohol withdrawal. Can you imagine the last thing you think before you die a horrible death from delirium tremens being how much you want Arby’s. Just shaky-poos and French Dips baby. F***** conk out to some Pink Floyd.” “I think she was high (from alcohol and weed) when she was making that Baby and she didn’t remember the next day, poor thing.”
Alcohol and nicotine (n=23)	Discussions pertaining to the use of multiple substances or the mention of multiple substances in the same tweet	“Ain’t nobody talking about giving pregnant women cigarettes and vodka I’m pro-choice and I’d cuss a woman out who was planning to carry a baby to term yet drinking alcohol I know all about birth defects my niece is a nurse my grandmother before her...That is not what we” “This is no different tNhan taking my kids with me into Walgreens, CVS, the supermarket where #alcohol & #tobacco are in abundance. Or eating Mexican dinner with family with margaritas. And I’m still sick. I was refused service. I’m utterly disappointed & outraged over this!”
Nicotine and cannabis (n=10)	Discussions pertaining to the use of multiple substances or the mention of multiple substances in the same tweet	“Smoking weed/cigarettes while pregnant was never said to be okay BUT moms do it anyway if they want to do THATS THEIR PROBLEM! STOP MAKING IT YOURS! If you got an opinion then okay but if someone else dont agree F*** IT! Why get so heated just cuz they feel different than you? 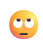 ” “There’s no one consistent in this house like our family’s middle child 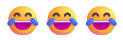 he wakes up smokes a cigarette, comes back and rolls a joint and goes to blaze it, he’ll roll another one before lunch and blaze it and another one & blaze it before supper and before sleeping.”

#### Subthemes

To extract more meaningful insights from each thematic area, we created additional subthemes within the identified main substance combinations. The most frequently reoccurring topics were lifestyle, perceptions of others’ use, legal ramifications, and public health. These prominent subthemes were based on the frequency of tweets in each category without necessarily pertaining to PPU. Broad definitions of each of the leading subthemes can be found in Table 8 along with and example tweets.

There are instances in which the subtheme breakdowns contained additional themes within them. The subtheme *lifestyle* contained more tweets with positive sentiment than negative sentiment (14 vs 12 tweets). The positive subtheme illustrated support for substance use when X users expressed personal experiences and normalcy with substance use. Meanwhile, the negative subtheme discussed how others’ use has affected the tweeters’ lifestyle choices. *Lifestyle* was the most frequently occurring subtheme within the major substance combinations, and thus, individuals were comfortable discussing their substance use through social media. Many individuals discussed daily substance use, social substance use, and family’s or friends’ substance use as it pertained to lifestyle decisions.

**Table 8:** Definitions and examples of the most frequently occurring subthemes connected to polysubstance use.

Subtheme and substance combination		Example tweet
**Lifestyle (n=38): content conveying certain habits and lifestyle choices pertaining to nicotine and alcohol**		
	Alcohol and nicotine	“In Stoke, the young ladies switch to menthol cigarettes when they are confirmed pregnant. And they increase their alcohol intake, as they are drinking for two.” “I don’t have debt...I don’t smoke & seldom drink alcohol. It impairs my judgement. Cigarettes & alcohol are expensive. You have to launder clothing more often which wears them out prematurely plus they cause health problems in kids. My dad smoked. I was sick as infant & kid.”
	Alcohol and cannabis	“Like 90% of my friends are married & starting families, instead of joining them in the baby making i’ve come to terms with that fact that i’m forever gonna be crazy aunt D with the cool tattoos that buys them alcohol & teaches them how to roll a perfect blunt & i’m ok with that.”
	Nicotine and cannabis	“There’s no one consistent in this house like our family’s middle child 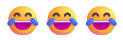 he wakes up smokes a cigarette, comes back and rolls a joint and goes to blaze it, he’ll roll another one before lunch and blaze it and another one & blaze it before supper and before sleeping.”
	Alcohol and drugs	“My favorite thing about not being pregnant is drugs & alcohol 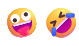 ”
**Perceptions of others’ use** **(n=33): discussions of substance use in others from witnessed events to assumed use**		
	Nicotine and cannabis	“Smoking weed/cigarettes while pregnant was never said to be okay BUT moms do it anyway if they want to do THATS THEIR PROBLEM! STOP MAKING IT YOURS! If you got an opinion then okay but if someone else dont agree F*** IT! Why get so heated just cuz they feel different than you? 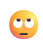 ”
	Alcohol and nicotine	“Ain’t nobody talking about giving pregnant women cigarettes and vodka I’m pro-choice and I’d cuss a woman out who was planning to carry a baby to term yet drinking alcohol I know all about birth defects my niece is a nurse my grandmother before her...That is not what we”
	Alcohol and drugs	“...like I know everything, I just can’t imagine how decestated [devastated] I would be personally to find out I had been unkowingly harming my baby. Whether it be pills/alcohol/illicit drugs babies aren’t intended to be exposed to any of it and if that was me I would want to consider abortion.”
**Legal ramifications (n=28): discussions of legal situations, such as laws, regulations, and charges**		
	Alcohol and nicotine	“If you can vote, die in battle, drive, & sadly enough have an abortion at 18 why not leave the tobacco law in place for 18 & possibly even lower alcohol age to 18 Just saying”
	Alcohol and drugs	“More talkin out of your backside because clearly you either haven’t read or understand the bill! There’s only a potential to be prosecuted for miscarriages if they are deliberate and willful like taking excess drugs and alcohol to deliberately cause a miscarriage.”
**Public health (n=23): content that looks to educate the public on substance use or offer support to users**		
	Alcohol and drugs	“Are you a #family member of someone with a #drug or #alcohol #addiction? Do you support a #partner, a #child, #parent or #friend with #substance misuse? Do you need #help but don’t know where to turn to? Call Michael on (phone #) for a friendly confidential #chat #carers #cope https://link”

Another common theme throughout *perception of others’ use* was “use while pregnant*.*” The total number of tweets in this subtheme of the major substance combinations discussed was 33. Additional themes allow for more accuracy and ensure that the exact topic discussed is being captured, further assisting the discovery of more specific recurring themes. The prevalence of the subsubtheme *perceptions of others’ use* can be attributed to the intrinsic purpose of social media platforms such as X. Oftentimes, these platforms are used for individuals to voice their opinions of disagreement and concession. Some users stood up for the pregnant women while still holding their stance that substance use should be avoided:

Smoking weed/cigarettes while pregnant was never said to be okay BUT moms do it anyway if they want to do THATS THEIR PROBLEM! STOP MAKING IT YOURS! If you got an opinion then okay but if someone else dont agree **** IT! Why get so heated just cuz they feel different than you?

Others chose to adhere to their beliefs and shame individuals who thought otherwise and even went as far as to suggest aborting the pregnancy if it was believed that substances were used while pregnant:

Whether it be pills/alcohol/illicit drugs babies aren’t intended to be exposed to any of it and if that was me I would want to consider abortion.

Discussions of abortion and miscarriage laws were the main topic behind the subtheme of *legal ramifications*. Criminal charges can be brought against a mother who uses substances while pregnant; some of those charges include child endangerment, attempted aggravated child abuse, child neglect, and even manslaughter [12]. Many of the tweets that fell under this subtheme were related to when a woman could be charged for a miscarriage if she ingested substances during her pregnancy. One discussion revolved around whether a woman would be held responsible for a miscarriage due to substance use if she was unaware of her pregnancy.

Users who are currently pregnant and struggling with substance abuse may find all the negativity and judgment discouraging, thus making them hesitant to reach out for help due to fear of persecution. Researchers who studied secrecy versus disclosure found several explanations for apprehension in pregnant women with substance use issues, specifically shame and fear of judgment as motives for not reaching out to family and physicians [11]. Mothers who use substances are ashamed of the harm they are potentially causing their children while they live in fear of criminal charges they could be subject to, along with the possibility of having their children taken away from them [12,27].

The following is one of many tweets discovered in our corpus that project negative opinions of women who partake in substance use during their pregnancy. It exemplifies the stigmas that surround the topic:

just really grossed out by girls who smoke weed, vape, and drink while they’re pregnant do better.

## Discussion

### Principal Findings

#### Quantitative Findings

The quantitative sentiment analysis revealed clear variations in user sentiments toward PPU. Tweets related to PPU presented a significantly lower percentage of positive sentiments compared to those unrelated to PPU. In addition, PPU-related tweets presented a significantly higher percentage of negative sentiments. Furthermore, neutral sentiments were significantly less frequent in PPU tweets—only 9.78% (474/4848) compared to 27.21% (1319/4848) in the non-PPU sample—highlighting a lower tendency to express neutral views on PPU. We also identified a set of complicated substance use combinations in the 4848 PPU tweets, among which paired substance use (4134/4848, 85.27%) dominated, with “alcohol and drugs,” “meth and drugs,” “weed and meth,” and “cocaine and drugs” as the top 5 paired substance types. The combination of “alcohol and drugs” use was ranked as the most frequently reoccurring combination in PPU discussions on X. A total of 11.12% (539/4848) of the PPU tweets mentioned 3 substances, and 3.61% (175/4848) included >3 substances.

#### Qualitative Findings

The results from thematic content analysis illustrated that alcohol and drugs were the driving forces of the substance combinations. These substances were the most frequent in the combinations discovered through the qualitative analysis. Alcohol is a widely accepted substance due to its use in social gatherings and personal consumption as a legal substance if users’ ages meet legal requirements, thus making it a leading substance in this study.

On the basis of the qualitative sentiment analysis, PPU had an overall negative sentiment in tweets, implying that substance use during pregnancy was mostly not supported in the gathered tweets. Except in specific situations, X users essentially perceived that PPU is dangerous and should be avoided. Some users even suggested abortion to avoid adverse outcomes for the infant. Consistent themes from this intersection include how others viewed the pregnant individual’s PPU regarding the negative health outcomes for the child when substances are used during pregnancy and the significant stigma related to using substances while pregnant. The subtheme *public health* provided some knowledge to educate pregnant individuals on avoiding, reducing, and intervening in PPU and offer support to this vulnerable population.

Furthermore, a high volume of tweets pertaining to *legal ramifications* (n=28) reflected impassioned speakers on social media platforms. Users will tweet their opinions to corroborate, advertise, and support their views on the current state of US politics and policies. One major topic dealt with miscarriage charges for pregnant individuals who used substances during the pregnancy and how their behaviors may have led to the miscarriage. This can be connected to the fear of disclosing their substance use patterns while pregnant.

### Comparison With Prior Work

Tweets containing shame and judgmental opinions of mothers who use substances were also found. This is consistent with previous work investigating the reasons for the secrecy of drug use in pregnant women [11]. Although the X platform is anonymous, these negative opinions expressed by X users may make it difficult for pregnant individuals to disclose their drug use and a desire for help for fear of being judged on a large social media platform. Public health agencies may want to ensure nonstigmatizing messaging with treatment resources to pregnant women on social media as they may routinely access the platform to gather information but may be less inclined to tweet about it. Our identified major substance combinations were consistent with those found in previous studies. However, while previous research has typically identified alcohol and cannabis or alcohol and tobacco as the most common substance combinations among pregnant individuals [1,28], our analysis revealed that alcohol and drugs were the most prevalent substances within the combinations studied. Through sentiment analysis, we were able to identify a narrow gap between positive sentiment and negative sentiment tweets; this can be due to the normalization of substance use as previous research on the use of alcohol and cannabis through X found a similar theme of normalization [26].

### Limitations

One limitation of this study was the fact that there were not many tweets specifically discussing PPU in the perinatal population, coupled with the lack of first-person experiences being shared. It has been noted that many pregnant individuals are afraid to admit substance use due to the associated stigma [11]. While X is a large social media platform that is used to anonymously interact with others on the web, few individuals openly disclosed personal use during pregnancy due to stigma, negative health effects, and potential legal ramifications. Next, although our list of inclusion terms was rigorously selected by clinicians, it may not have been exhaustive, potentially missing some relevant terms and tweets. Moreover, with the recent X data access policy changes, without substantial funding and resources, public social media data such as those of X cannot be continuously accessible and affordable to social media researchers, including those who conduct health care social media research. In addition, it is challenging to study different types of X users, who are not explicitly categorized into organizations or any other user groups, without a high level of self-disclosure by users themselves.

### Conclusions and Future Directions

Among the substance combinations discovered through the qualitative analysis, we identified alcohol and drugs as the most prevalent substances discussed in PPU tweets. The legality of alcohol use and the growing legalization of marijuana could be the reason for the increased frequency of pairing these specific substances. We found that the public opinion of X users was generally that pregnant individuals should avoid using single or multiple substances. Our quantitative and qualitative analyses supported more negative sentiment discussions of PPU on X, with additional communication exchanges discussing criminal charges for pregnant women who use drugs. It is promising to see public health messaging by individuals and public health organizations on the benefits of good prenatal care, recommending avoidance of substance use during pregnancy and offering support to the vulnerable perinatal population. In addition, substance treatment resource information was exchanged on where and how to seek assistance for individuals or family members who were addicted to drugs. Retweeting these types of positive communication exchanges could provide an opportunity for broader dissemination and education of individuals on ways to acquire these needed resources. As such, social media such as X could be further used by health organizations and web-based communities to educate the perinatal population and provide needed support and help anytime, anywhere, and at scale.

Overall, this research allowed us to gain a more holistic understanding of PPU patterns discussed on X, uncovering the complexity of the alarming PPU phenomenon and its important implications to PPU practitioners and associated public health policy makers regarding legal ramifications. Through a mixed methods approach, this study established a solid foundation that requires further investigations to understand the underlying mechanisms of PPU contextually, technically, and clinically. The methodologies used in this study can be adapted to analyze content on other social media platforms such as Reddit, Instagram, TikTok, Pinterest, and Weibo, where discussions about substance use also occur [29]. From a computational analysis perspective, we plan to train ML models to automatically detect the PPU tweets at scale, potentially identifying at-risk women who use substances and, thus, providing them with the proper knowledge, early intervention, treatment for PPU-related addiction, and necessary support that they might not have otherwise obtained. There remains a significant need to advocate for and implement PPU-related early intervention programs at the individual, community, clinical, and governmental levels. This study implies that providing relevant patient education through early intervention programs, access to addiction treatments and community support, and extended perinatal care services is critical to supporting the vulnerable perinatal population with PPU for better health outcomes for both mothers and babies. In addition, this study did not cover PPU for medical purposes, which can be our future research if we have more access to PPU-related clinical data to supplement our social media data to further enrich our knowledge and the clinical implications in this important health domain.

## Appendix

Multimedia Appendix 1 Keywords provided by our clinical team for this research.

Multimedia Appendix 2 Overall substance combinations in the perinatal polysubstance use tweets and the top 60 substance combinations labeled as other.

## Abbreviations

ML: machine learning
NLP: natural language processing
PPU: perinatal polysubstance use
PUD: polysubstance use disorder
RQ: research question
SUD: substance use disorder
VADER: Valence Aware Dictionary and Sentiment Reasoner
