# Supplementary material for: Uncovering the Complexity of Perinatal Polysubstance Use Disclosure Patterns on X: Mixed Methods Study
Source: J Med Internet Res. 2024 Sep 20;26:e53171. doi: 10.2196/53171 (PMC11452753; doi:10.2196/53171)
Supplement: Multimedia Appendix 1 [file jmir_v26i1e53171_app1.docx]

## Appendix 1

**Keywords provided by our clinical team for this research:**

- **Substance-related keywords:**

'Alcohol', 'Cocaine', 'Amphetamines', 'methamphetamine', 'Hallucinogens', 'nicotine', 'Opioid', 'sedatives', 'diazepam', 'Tobacco', 'SUD', 'Heroin', 'Cannabis', 'MDMA', 'LSD', 'weed', 'meth', 'XTC', 'benzodiazepines’, 'stimulants', 'Morphine', 'Fentanyl', 'Codeine', 'Oxycodone', 'Oxymorphone', 'Hydrocodone', 'Norco', 'Ritalin', 'Adderall', 'Xanax', 'Valium', 'marijuana', 'cigarette', 'vaping', 'vape', 'e-cigarette', 'ganja', 'synthetic cannabinoids', 'spliff', 'kush', 'sativa', 'indica', 'chronic', 'blunt', 'hydro', 'skunk', 'reefer', 'joint', 'dabs', 'shatter', 'budder', 'wax BHO', 'butane honey oil', 'hash oil', 'edibles’, ‘weed cookies', 'space cake', 'pot cookie', 'pot brownie','medibles','spice','K2','chminaca','AB-FUBINACA', 'synthetic weed']

- **Prenatal and child health-related keywords:**

[Pregnancy,’‘pregnant,’’preggers,’’baby,’’prenatal,’’obGyn,’’childbirth’]
