# Supplementary material for: Uncovering the Complexity of Perinatal Polysubstance Use Disclosure Patterns on X: Mixed Methods Study
Source: J Med Internet Res. 2024 Sep 20;26:e53171. doi: 10.2196/53171 (PMC11452753; doi:10.2196/53171)
Supplement: Multimedia Appendix 2 [file jmir_v26i1e53171_app2.docx]

## Appendix 2


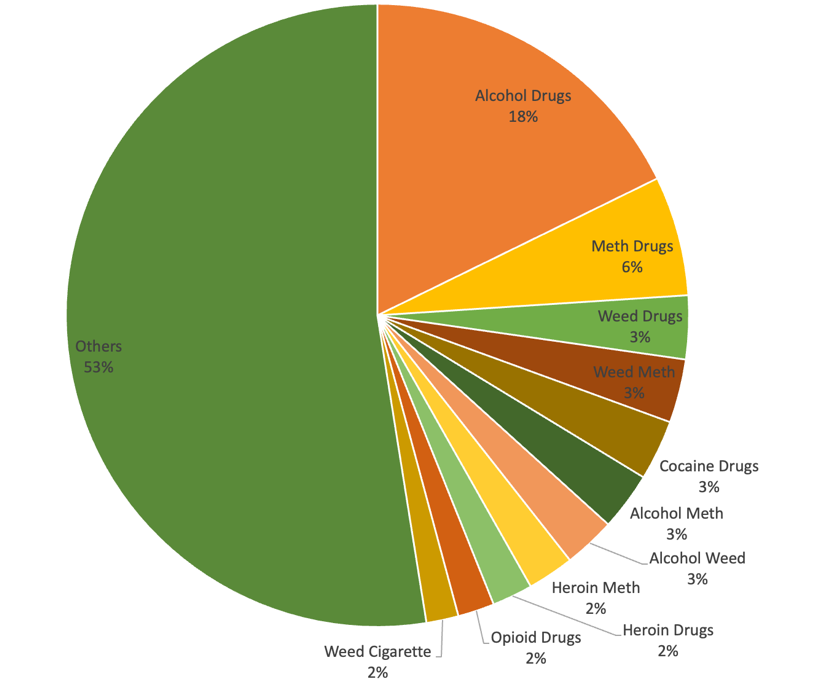


Figure 1. Overall Polysubstance Combinations in the PPU Tweets

Table 1. Lists the Top 60 Polysubstance Combinations Labelled as ‘Other’ in Appendix 2, Figure 1

| Polysubstance Combinations | Percentage | Polysubstance Combinations | Percentage | Polysubstance Combinations | Percentage |
| --- | --- | --- | --- | --- | --- |
| vaping & vape | 2.04% | drug & Coke | 0.74% | Adderall & drug | 0.52% |
| Opioid & drug | 1.90% | Alcohol & Heroin | 0.72% | Xanax & drug | 0.52% |
| marijuana & drug | 1.86% | Hydrocodone & hydro | 0.72% | meth & Coke | 0.52% |
| weed & cigarette | 1.67% | Alcohol & Opioid | 0.70% | meth & cigarette | 0.50% |
| Fentanyl & drug | 1.63% | Tobacco & cigarette | 0.70% | Alcohol & Cocaine & drug | 0.50% |
| Cocaine & meth | 1.55% | weed & Coke | 0.68% | Cocaine & Coke | 0.50% |
| Alcohol & cigarette | 1.46% | meth & Fentanyl | 0.68% | SUD & drug | 0.50% |
| methamphetamine & meth | 1.30% | cigarette & vaping | 0.66% | meth & vaping | 0.47% |
| Alcohol & Cocaine | 1.24% | Heroin & Fentanyl | 0.64% | Tobacco & vaping | 0.47% |
| drug & hydro | 1.22% | nicotine & vaping | 0.60% | Tobacco & drug | 0.45% |
| weed & edibles | 1.18% | Heroin & weed | 0.60% | stimulants & drug | 0.45% |
| Alcohol & marijuana | 1.09% | Alcohol & Cannabis | 0.60% | Alcohol & meth & drug | 0.43% |
| weed & vape | 0.93% | Opioid & Heroin | 0.60% | weed & skunk | 0.43% |
| Alcohol & Tobacco | 0.91% | Cocaine & Powder | 0.58% | Tobacco & vape | 0.43% |
| Cocaine & Heroin | 0.89% | Alcohol & Coke | 0.58% | Opioid & chronic | 0.41% |
| Cannabis & edibles | 0.83% | Cocaine & weed | 0.56% | drug & chronic | 0.41% |
| meth & Marijuana | 0.78% | cigarette & drug | 0.56% | cigarette & e-cigarette | 0.41% |
| cigarette & vape | 0.78% | Cannabis & meth | 0.56% | Cocaine & Heroin & meth | 0.39% |
| Opioid & meth | 0.76% | Alcohol & drug use | 0.52% | Alcohol & Nicotine | 0.39% |
| Cannabis & drug | 0.76% | Alcohol & drug & drug use | 0.52% | Cannabis & vape | 0.39% |
